# Supplementary material for: Experiencing the COVID-19 pandemic as a healthcare provider in rural Dhanbad, India: An interpretative phenomenological analysis
Source: PLoS One. 2022 Aug 25;17(8):e0273573. doi: 10.1371/journal.pone.0273573 (PMC9409569; doi:10.1371/journal.pone.0273573)
Supplement: S1 Appendix — (DOCX) [file pone.0273573.s001.docx]

**S1 Appendix. Significant utterances of each participant and annotations of non-verbal cues that underpinned the interpretational phenomenological analysis**

**Varun**:

“We only have one bedroom, so to avoid passing the infection to my husband, I started sleeping in the kitchen since the pandemic started. I have not been in the same room as my husband for so many months now. I want to be with him but at the same time I don’t want to put him in danger.”

“Day before yesterday was so bad that I did not even have time to pee. I had breakfast before coming to work, and then I had one glass of tea at 8 `o clock at night, then went back to the ICU. No water, no toilet break. I worked all night as well, and I lost 4 patients. It was the worst day of my life. There was nobody to relive me, I worked alone for 30 hours before a relieving doctor came!”

“We are trained to do everything we can for a patient, but our hospital has decided not to do CPR on patients because of the aerosolization risk. I can’t in good conscience say that I have done everything possible. I feel like I am not doing the best I can”

“I follow all the treatment guidelines, I read up on research, I do everything to the letter, but patients still die. I can’t figure out why, and I can’t live with that. I need to know what I could have done better. I need to know what I got wrong, why did my patients die.”

“The people were generally very grateful. They are always saying oh doctor you are our only hope and this and that. But when I mentioned the cost of the drugs, they saw me like a thief. It was very much visible from their faces though they did not say much. (Long pause). Actually, that is not true, a patient’s family members kept on calling me one after the other and said I am there only to suck their hard-earned money. (Pause). Still those words disturb me. Am I setting the rate of the medicine? I am also equally pained when my patients die because they cannot afford the medicine, and I am tired of fighting with my management over the unfairly high bed costs, but ultimately, am I responsible for the cost? You tell me!”

“I wish there was someone I can share my caseload with. But no such luck. I have been working alone and I don’t think I will be getting anyone to work with anytime soon. There is no money to hire another doctor, that’s what I have been hearing since months.”

“When I see anyone venturing out without a mask, I give them a piece of my mind. We are risking our lives, and they’re putting us at more risk unnecessarily. I just get so angry…let’s not talk about this only. Sometimes I think why should I work when people are so careless about their own safety.”

“There is nothing better than watching a patient walk out of the hospital. For a few minutes I forget all about the not getting break to eat or go to the toilet and not getting any help and rest and I just feel good watching them reunite with family and go home. This is why I became a doctor after all.”

“While working, there is no time to think, it is like I am on autopilot. I take a long bath when I go home and during the bath, I talk to myself, I tell myself that this war is not over, you may have lost today but tomorrow will be yours, now go and sleep and get ready for tomorrow. It sounds crazy, but it really works for me.”

“We all need support. Mental wellbeing, especially among doctors is taboo. We see it as a sign of weakness. But this pandemic has taught us that even the best of us needs a helping hand now and then. (Pause). We need counselling, time off to spend away from hospitals and patients, and more support…they should hire more doctors so that we treating doctors can work reasonable shifts and get enough rest.”

**Vayu:**

“Every time I get a call, I just pray that it is not shortness of breath. You see, I have diabetes, so I am really vulnerable to COVID. But if I don’t work, who will feed my family…I don’t have a choice.”

“We get a small portable oxygen cylinder that lasts maybe 1 hour when we go to see a patient. After that we have to shift to the ambulance, and the cylinder in the ambulance lasts maybe 6 hours. But we are not allowed to shift the patient to the ambulance unless the family has a bed confirmed at a hospital. But there is so much shortage of beds. (Long pause). You know, too many people have died after the portable cylinder ran out. I have seen this so many times now. Sometimes, I have to wait outside the hospital. Some lucky patients got a bed before the cylinder in the ambulance became empty, but so many unlucky patients did not get bed on time. Their deaths (pause)…the deaths…they haunt me all the time. So many people have died on my watch that now I feel everything is pointless, my work, my life, everything.”

“Sometimes when I reach to a patient’s home, they are already so far gone, I know they probably will not even reach the hospital alive, on the way itself they will go. But the family ask me to do something, but I am so helpless (pause)…there is nothing to do.”

“I was transporting a patient to the hospital. As there was no beds, I was asked to wait outside the gates, along with many other ambulances. After 2 hours, one by one the ambulances were asked to come in as beds became available. Our turn came after 4 hours. The patient’s son started shouting at me after sometime. He said all this was just drama so that the hospital can ask for more money. He was non-stop abusing me; I did not throw him out only because his mother needed the oxygen to live. I thought my pride was not as important as another person’s life.”

“My break is during the drive, and the driver takes a break when I assess the patient. We have no dedicated breaks otherwise. I have forgotten what it is like to stop at a tea stall and drink tea biscuit, chatting with the tea stall guys. That used to be so normal for me, I made friends of the tea stall owners and the cleaning boys and all. I used to drink so many teas and get enough breaks. The driver also. But now it is a luxury. We just stop for tea and take it away on the ambulance.”

“I hand out masks to those who don’t wear them, and I don’t get out of their way till they wear it. Some people have been annoyed by it, but I don’t care. We are playing with our lives and they are roaming as if they don’t care, I will not have any of that nonsense.”

“When I reach a patient on time and when they reach to the hospital safely and get a bed, I feel very happy. They at least get a real chance to fight. I have done my job then I feel.”

“I don’t know…I wish to hug my wife, but I don’t want to infect her. So, I sleep outside. I just see her and my family safe inside the house and tell myself that if they have to be safe, I have to be outside and I have to be alive. I need to be there for them. If I am gone, who will feed them? This one thought is enough, I get the energy to sleep outside and stay away from my wife and follow all the COVID protocols that the government has put in place while working with patients.”

“Whatever capacity increase has happened, it has all been in the cities. We have been forgotten, but sadly, COVID hasn’t forgotten us. COVID does not know that this is a city, this is where the have all the beds and all the medicines and everything, so let me come here and leave the villages alone. It will spread everywhere.”

**Sani:**

“I always keep thinking, what if I bring it home from the hospital. My old mother lives with me. I am always afraid of being in the same room with her. I work with so many patients and we don’t know if this virus survives on clothes. Even with PPE, of course some part of my clothes may be exposed. (Pause). What if the virus gets attached to my clothes and I bring it home and my mother is affected? This is my constant worry. But…I have chosen this work, now I shouldn’t complain”

“There are no specialists here, I have to be everything that my patients need me to be. But I can’t stop thinking that maybe because I volunteered, my patients are getting a poorer quality of care. Maybe if I didn’t volunteer, the government would have sent a specialist, and they would have done a much better job. They are trained for these things, I am not.”

“If I have a doubt, there is nobody to ask. I have a to phone a friend, and if they don’t pick up, try another one. If they tell me to do something which I have not done in a very long time, then that is too bad, because only I have to do it, I cannot refer to someone else. But of course, I have volunteered now so I have to do my best and leave the rest to God. But you know, all the doubts and frustration are gone when I am signing the discharge papers and the patient gets to go home to their family. It gives me a sense of fulfilment.”

“We don’t have enough drugs; we have to ration them. I feel like I am playing God, choosing who gets the drugs and who doesn’t. It is not right. (Pause). It is just not fair. (Sobbing). I don’t want to do this but I am forced to. I should at least make sure that the drugs are going to the ones who need it the most. (Pause). But its not easy denying someone for a life saving drug just because I have decided they don’t have a chance.”

“When I volunteered, I thought I will be just assessing patients and sending severe ones to the hospital. But there are no beds, and I am treating the severe ones also. How is my care going to be good? The patients I have lost, maybe if they went to a trained specialist, they might have survived…who knows?”

“Will this end? I just can’t see an end to all this suffering…God only knows when this will end. There is nobody to lean on. Just me and a nurse and a midwife. And so many patients! Just don’t have any time for anything other than seeing the patients. I have thought of quitting so many times. But it would be irresponsible of me to quit now, when there is so much need. Every day at prayer time, we have the habit of reading a verse from the Ramayana. When I read the verse, I remember that despite so many struggles if Prabhu Ram can be so calm and perform his duties, I too must learn from him and do my duty. I forget about my doubts and about quitting and just go to work.”

“When I volunteered to treat, a close friend said, oh so you have joined the goldrush. I felt so disappointed that even people who know me are thinking like this. As if I am some vulture waiting for bad things to happen to people so that I can profit from it…this is what entire society is thinking about doctors now. Of course the bills that the hospitals are charging are horrible. When I saw the amounts on the news, I was so disgusted, but we all are not like that. I volunteered because I felt my village needs me and I’ve been working hard sincerely, but we all get painted with the same brush.”

“Don’t they see how many people are dying? I really don’t understand why they are so stubborn to cost more lives. They should be put in jail.”

“Why cant they give special permission to overseas trained doctors to practice without the MCI exam? They can be supervised by a Indian licensed doctor even. And make a rule that they can work without the MCI exam only in rural and suburban areas. Our staffing problems will be gone.”

**Bhumi:**

“I think I have developed OCD, I wash my hands too many times. I know all my patients have COVID, but I don’t want to cross-infect anyone. Some may have lesser virus load than others, so if I cross-infect their load may increase and their health may become worse. I am very careful about this.”

“We get our supplies of oxygen and PPE and drugs and everything only after all the metropolitan hospitals get their share. Whatever is left has to be shared among many hospitals in the semi urban and rural places. We always get short-changed. Our needs are underestimated. (Pause). If we ask for more, we never get it on time. We are like Cinderella and the hospitals in the metropolitan areas are like Cinderella’s stepsisters and the government is the stepmother. They are thinking that all patients who have severe infection will anyhow go to the city only, so we must stock up the city hospitals with drugs and oxygen and everything, rural hospital is anyway only for triage. Okay, that approach worked in the first wave. But in this wave, the city hospitals are full, so all rural patients are staying in the rural hospitals. (Pause). The government’s approach to distributing resources has not changed to meet this reality. They are still thinking rural patients will anyway come to the city.”

“We cannot touch the patients without wearing full PPE. But touch is a very big part of healing, it shows the patient that we care. But now with full PPE, if I smile also the patient does not know. Now everything feels mechanical. It is not the way to care for the patients.”

“(Audible long exhalation). The patients’ families are thinking we are looting them. The way they talk to us, it is clear they think like this. Fact is, I have not even received a raise in the past two years. Even with such an increased patient load, I am drawing the same salary. (Pause). Still, I am doing my job with the same commitment without complaining. So, I don’t know why I am being seen as the enemy.”

“It makes me so mad. I have stopped them on the road and had arguments. My husband thinks I am going to annoy someone so much that they might hit me, he always asks me to stop. But seeing so many deaths, I know I will never stop arguing about masks and social distancing.”

“I have no rest. My whole body aches, but I have to go on because there is nobody else to do my job. I don’t get paid enough to work like this, but our COVID warriors need me. If I put leave, their work with increase so much that they will have to ask the family members only to look after the patients. But the patients don’t even thank us when they get better. They thank only the doctor. We also do a lot of hard work. Why don’t we get recognized as much? In fact, we spend more time with the patients and their family members than the doctors, then they should be thanking us more, instead they thank the doctor and go away. Is my work so worthless? I feel worthless.”

“I am not coping at all actually. (Audible sigh). Right now, there are so many patients that I am not getting time to process what is going on. So, I think my coping strategy is to bundle all these emotions and keep on the back burner and just do what I am told. Someday the time will come to address this and, on that day, mostly that day will be the day this pandemic ends, I am sure I am going to have a nervous breakdown.”

“The idea to involve medical and nursing students in the care of COVID patients is a good idea. But if you see, there are very few colleges outside cities. We continue to be short staffed. The government needs to do something to bring more doctors and nurses to the rural areas. Maybe if we can get paid more, we will also be happy and more people will also be willing to work here. We are after all risking our lives, we should get a bonus and salary hike. I think we earned it.”

**Agni:**

“I follow all the guidelines for COVID safety. But you never know…anything can happen. I don’t think anyone really knows a lot about this COVID virus, so, even if they tell one thing is safe, like say they told COVID will not live on hair. How I can be sure that they know for sure? So, I like to take extra precautions. I wear plastic bags on my shoes in the hospital and I throw those covers before coming home. I wear hair covers. I think you can’t be too careful with this.”

“Sometimes I feel as if I am as clueless as my patients’ family. I know all the government guidelines, but I still can’t explain to the patient why someone gets badly infected and someone does not get symptoms also. I don’t know why even after giving the correct dose of Remdesivir the patient still dies. When they ask me why this happens, I feel clueless. But I don’t think I am alone, everyone is clueless about COVID”

“I have seen so many people dying alone, without any loved one near them in their last moments. Their families plead with us to let them be with the patient at the end. The hopelessness that I feel at these times, I have never felt it before in my life. I can’t let them be with the patient as it is against the guidelines, but as a human I also don’t want anyone to die alone. It is the saddest part of my day.”

“This pandemic has gone on for too long, work has become boring. But these patients are my responsibility. The only thing that keeps things going is the fact that I know that I am making a difference to them.”

“Outwardly, everyone are acting like they are grateful to healthcare workers, but inside they all think we are looting them. Because in the news they have shown how hospitals are overcharging people even more than the government norms, this has made a very bad impression on people.”

“I have called the police on people who don’t wear masks. They should be booked under the pandemic act so that they learn a lesson and others also get deterred from walking without masks.”

“The patients need us right now; we have to be strong. But the truth is, I am scared. I don’t know what will happen. I am unable to sleep properly. I worry a lot, but when it is time to go for duty, I just shut off my mind and go. Once duty is over, I worry again. I talk to my family, but end of the day, no matter how they try to help me, no matter what they say, it is me who is risking my life. Nobody can understand my fear and what I am going through. And if I want to talk to my peers who will understand my situation because they are in the same boat, they are too busy. Plus…(long pause) if I say I am afraid, even if they themselves are afraid, they will see me as weak and unworthy of working in healthcare.”

“I want to take some time to recover, but my leave was not approved because there is no replacement. I have to work even though I think I have burnout. I just want some days off to refresh and I will work with double enthusiasm, but its not possible as there is no replacement.”

“I don’t need to be treated like a hero, just want to be treated like a human being. I also have a family, I also need rest.”

“I just want to be safe, for that we should get enough PPE, I want rest, for that the government should increase beds in rural hospitals and hire more staff, and I want better pay, for that our managements must recognize our efforts and give us a bonus for working in this pandemic and risking our lives.”
