# Supplementary material for: Experiencing the COVID-19 pandemic as a healthcare provider in rural Dhanbad, India: An interpretative phenomenological analysis
Source: PLoS One. 2022 Aug 25;17(8):e0273573. doi: 10.1371/journal.pone.0273573 (PMC9409569; doi:10.1371/journal.pone.0273573)
Supplement: Attachment — Submitted filename: Response to Reviewers.docx [file pone.0273573.s002.docx]

**Response to reviewers**

Below are our point-by-point responses to the questions and comments raised during peer review. The questions and comments are in black font and our responses are in blue font.

Please note: The page and line numbers mentioned in this document pertain to the manuscript with changes tracked, in the all-markup view. Additionally, the reference numbers are different in this document and in the manuscript; the reference numbers in this document correspond to the order in which the references are cited in our responses herein.

**Comments from Academic Editor:**

1. When submitting your revision, we need you to address these additional requirements.

Please ensure that your manuscript meets PLOS ONE's style requirements, including those for file naming. The PLOS ONE style templates can be found at 
<https://journals.plos.org/plosone/s/file?id=wjVg/PLOSOne_formatting_sample_main_body.pdf> and 
<https://journals.plos.org/plosone/s/file?id=ba62/PLOSOne_formatting_sample_title_authors_affiliations.pdf>

**Response**:

Thank you for considering our manuscript; we can affirm that our manuscript meets PLOS ONE’s style requirements, including the ones for file naming.

2. Please note that in order to use the direct billing option the corresponding author must be affiliated with the chosen institute. Please either amend your manuscript to change the affiliation or corresponding author, or email us at [plosone@plos.org](mailto:plosone@plos.org) with a request to remove this option."

**Response:**

We have emailed [plosone@plos.org](mailto:plosone@plos.org) requesting to remove the direct billing option.

3. In your Data Availability statement, you have not specified where the minimal data set underlying the results described in your manuscript can be found. PLOS defines a study's minimal data set as the underlying data used to reach the conclusions drawn in the manuscript and any additional data required to replicate the reported study findings in their entirety. All PLOS journals require that the minimal data set be made fully available. For more information about our data policy, please see <http://journals.plos.org/plosone/s/data-availability>.

**Response:**

We have now included an appendix containing all the significant utterances by each participant during their interview; based only on these utterances, we proceeded with our analysis. The appendix may thus, be deemed the minimal dataset. As our study is a qualitative study, and identifying information, including the names of the hospitals where the participants are employed, residential location, and names of participants’ family members and some of the patients under the care of the participants, is present in the raw data, we are unable to deposit it in a publicly available repository. We will make the raw data available upon request. The request for the raw dataset can be directed to:

The Secretary,

Sri Jagannath Healthcare and Research Center - Independent Ethics Committee,

Steel gate, Main road, near hero showroom, Dhanbad, Jharkhand - 828127
sweta.sjhrc@gmail.com

4. Upon re-submitting your revised manuscript, please upload your study’s minimal underlying data set as either Supporting Information files or to a stable, public repository and include the relevant URLs, DOIs, or accession numbers within your revised cover letter. For a list of acceptable repositories, please see <http://journals.plos.org/plosone/s/data-availability#loc-unacceptable-data-access-restrictions>. Any potentially identifying patient information must be fully anonymized.
   Important: If there are ethical or legal restrictions to sharing your data publicly, please explain these restrictions in detail. Please see our guidelines for more information on what we consider unacceptable restrictions to publicly sharing data: <http://journals.plos.org/plosone/s/data-availability#loc-unacceptable-data-access-restrictions>. Note that it is not acceptable for the authors to be the sole named individuals responsible for ensuring data access.

We will update your Data Availability statement to reflect the information you provide in your cover letter.

**Response:**

We have now included an appendix containing all the significant utterances by each participant during their interview; based only on these utterances, we proceeded with our analysis. The appendix may thus, be deemed the minimal dataset. As our study is a qualitative study, and identifying information, including the names of the hospitals where the participants are employed, residential location, and names of participants’ family members and some of the patients under the care of the participants, is present in the raw data, we are unable to deposit it in a publicly available repository. We will make the raw data available upon request. The request for the raw dataset can be directed to:

The Secretary,

Sri Jagannath Healthcare and Research Center - Independent Ethics Committee,

Steel gate, Main road, near hero showroom, Dhanbad, Jharkhand - 828127
sweta.sjhrc@gmail.com

**Comments from Reviewer#1:**

1. General Points
This is an excellent and very interesting paper which provides a unique perspective regarding the experiences of healthcare workers in a rural setting – contrasting much of the current literature which focuses upon larger urban / teaching hospitals. It highlights the challenges associated with facing the COVID-19 pandemic in a severely under-resourced setting, which will be useful for the provision of support in future similar crises.
Have suggested minor clarifications – particularly for readers who are not familiar with the culture or rural healthcare environment.

**Response**:

Thank you for your kind evaluation of our work; we hope we have satisfactorily replied herein to all your questions and comments

2. Abstract

a. Suggest correcting second sentence to: ‘it has led to a myriad of health problems’

**Response**:

Thank you for pointing this out. We have rephrased the aforementioned statement to bring more clarity.

**Old text-**

“It has led to myriad mental health problems”

**Modified text-**

“It has led to a myriad of mental health problems” (Page No: 2 , Line No: 22)

b. Clarification of sampling method (see longer comment under methods heading)

**Response**:

Since IPA studies are concerned with understanding particular phenomena in particular contexts, the participants were selected purposively (rather than through probability methods) so that they can offer an insight into the phenomena of interest. Potential participants were contacted via referral: during the pandemic, the authors collaborated with the public health authorities of Dhanbad, India to investigate whether the frontline workers in different settings of the district could be supported in any way in their fight against COVID-19; as part of this endeavour, for this study, we asked healthcare professionals in our known circles to recommend the names of their colleagues working in rural areas of Dhanbad district; we then contacted them and informed them about the study and asked them if they were willing to participate. Once they gave informed consent, and their long-form interview was concluded, we asked them to recommend other healthcare professionals in their know who are also working in the same rural area, but are from a different health profession. Therefore, we have called our sampling method purposive heterogenous snowball sampling. We were not able to modify the abstract to reflect this due to the word count restrictions.

c. Suggest correcting to “participants were under mental duress” or “suffered from mental duress”

**Response:**

Thank you for pointing this out. We have rephrased the aforementioned statement to bring more clarity.

**Old text-**

“Our findings demonstrate that the participants were mental duress due to heavy workloads”

**Modified text-**

“Our findings demonstrate that the participants were under mental duress due to heavy workloads” (Page No: 2, Line No: 32–33)

d. Could highlight uniqueness of findings and study setting more in the abstract

**Response:**

Thank you for the suggestion. To elaborate on the uniqueness of the findings and the setting of the study in the “**Abstract**” section, the **“Conclusions”** subsection has been modified as follows.

**Modified text-**

This study is unique in the sense that not many studies have been conducted to evaluate the psychological issues of Indian HCPs during this pandemic. Much less is known about the mental health of HCPs in rural settings. Moreover, novel findings such as negative social perception of HCPs during the pandemic and HCPs resorting to spirituality as a coping strategy against stress, open a plethora of research opportunities wherein the results of this qualitative study, along with the existing literature and findings of future quantitative studies, can establish better understanding of the impact of the pandemic on HCPs. (Page No: 2–3, Line No: 41–47)

3. Introduction

a. Could consider elaborating on the challenges faced in the rural setting for readers not familiar with this environment

**Response**:

Unfortunately, very few peer-reviewed studies have been conducted on HCPs in the rural settings of India, their psychological issues, the challenges they come across as compared to those in the urban settings. So, we could not elaborate much on this, and whatever we add will be anecdotal, which we felt is not suitable for a scientific journal. However, a few challenges unique to the rural setting were uncovered during the study, like the resource distribution disparity, and we have elaborated on these themes in the **“3. Results”** section. We have however, added a few lines about the setting of the study in the **“2. Methods”** section under the subsection, **“2.3 Setting and Data acquisition”** as follows:

The study is set in the rural areas of Dhanbad, India. Dhanbad is a district in the Indian state of Jharkhand. According to the 2011 census, Dhanbad district has a population of 2,684,487 individuals, 53% of whom are male; the literacy rate is 75.71%, the poverty rate is 26.76%, and 80.07% of the population is Hindu [1]. Jharkhand has a doctor–patient ratio of 1:8200 [[2](https://www.dailyrounds.org/blog/this-state-last-got-a-new-medical-college-50-years-back/)]. (Page No: 6, Line No: 125–128)

4. Methods

a. Please clarify what you mean by purposive heterogenous snowball sampling – your explanation in the manuscript sounds like purposive sampling rather than snowball sampling (where initial participants identify further suitable participants).

**Response**:

As IPA studies are concerned with understanding particular phenomena in particular contexts, the participants were selected purposively (rather than through probability methods) so that they can offer an insight into the phenomena of interest. Potential participants were contacted via referral: during the pandemic, the authors collaborated with the public health authorities of Dhanbad, India to investigate whether the frontline workers in different healthcare settings of the district could be supported in any way in their fight against COVID-19; as part of this endeavour, for this study, we asked healthcare professionals in our known circles to recommend the names of their colleagues working in rural areas of Dhanbad district; we then contacted them and informed them about the study and asked them if they were willing to participate. Once they gave informed consent, and their long-form interview was concluded, we asked them to recommend other healthcare professionals in their know who are also working in the same rural area, but are from a different health profession. Therefore, we have called our sampling method purposive heterogenous snowball sampling. We have updated the explanation in the section “***2.2 Participants”*** of the manuscript to state with more clarity that we have used purposive snowball sampling.

**Old text-**

Through purposive heterogenous snowball sampling, five HCPs in rural Dhanbad, India, were informed about the study, all of whom gave written informed consent to participate.

**Modified text-**

Through purposive sampling, one HCP in rural Dhanbad, India, identified via referral from HCPs known to the authors, was informed about the study, and written informed consent to participate was obtained. The participant was then asked to identify another suitable HCP working in rural Dhanbad, India, but belonging to a different healthcare profession to their own, for inclusion in the study. In this way, through purposive heterogenous snowball sampling, five HCPs in rural Dhanbad, India, were informed about the study, and all of them gave written informed consent to participate. (Page No: 6, Line No: 114–122)

b. How did you identify the 5 participants?

**Response**:

As mentioned above, we asked healthcare professionals in our known circles to recommend the names of their colleagues working in rural areas; we then contacted them and informed them about the study and asked them if they were willing to participate. Once they gave informed consent, and their long-form interview was concluded, we asked them to recommend other healthcare professionals in their know who are also working in the same rural area, but are from a different health profession. Therefore, we have called our sampling method purposive heterogenous snowball sampling. We have updated the explanation in the section “***2.2 Participants”*** of the manuscript to state with more clarity that we have used purposive snowball sampling. We hope our revision is able to satisfactorily answer your question.

**Old text-**

Through purposive heterogenous snowball sampling, five HCPs in rural Dhanbad, India, were informed about the study, all of whom gave written informed consent to participate.

**Modified text-**

Through purposive sampling, one HCP in rural Dhanbad, India, identified via referral from HCPs known to the authors, was informed about the study, and written informed consent to participate was obtained. The participant was then asked to identify another suitable HCP working in rural Dhanbad, India, but belonging to a different healthcare profession to their own, for inclusion in the study. In this way, through purposive heterogenous snowball sampling, five HCPs in rural Dhanbad, India, were informed about the study, and all of them gave written informed consent to participate. (Page No: 6, Line No: 114–122)

c. Could expand on how you defined data saturation – did this occur as interviews were ongoing or as a review process after 5 participants were recruited?

**Response**:

We adopted an individual-oriented approach to data saturation [3]. We defined data saturation as when the same information began to be repeated by a participant without much change in non-verbal cues associated with that information. We continued probing a participant until data saturation was reached and then proceeded with the next interview. We have updated this in the **“2. Methods”** section under the subsection **“2.3 Setting and Data acquisition”** as follows:

We adopted an individual-oriented approach to data saturation [3] and defined data saturation as the point at which the participant began repeating the same information without much change in the non-verbal cues associated with that information. (Page No: 7, Line No: 150–153)

5. Results

a. I think the results of this study are very rich and tell a fascinating story, providing novel insights into pandemic management in a rural setting.

**Response**:

Thank you very much for the kind comment.

b. 3.2.1 c. This is a very interesting data subtheme – would it be worth expanding about general views of society regarding HCW prior to the pandemic? Did this change?

**Response**:

Thank you for the insightful query. Unfortunately, there are not enough studies to conclusively establish the general views of the society regarding HCWs in the pre-pandemic setting, and we did not probe the participants regarding the same. Therefore, it is difficult to do a comparative analysis of how or if the perception of the society about HCWs has changed during the pandemic. This is a limitation of our study and we have now discussed this in the **“4. Discussion”** section as follows:

Another limitation of this study is that although our study yielded a significant and novel finding regarding the negative social perception of HCPs during the pandemic and it would be worth exploring the general views of society regarding HCPs before the pandemic and how this subsequently changed during the pandemic, we were unable to conduct such an analysis as there are no studies to conclusively establish the views of the public regarding the HCPs prior to the pandemic, and we failed to probe our participants about how they felt they were perceived prior to the pandemic. The impact of the COVID-19 pandemic on the social perception of HCPs is an interesting future avenue of research with important implications for the doctor–patient relationship. (Page No: 32–33, Line No: 682–690)

c. Can you clarify what you mean by ‘stepmotherly treatment by the government’?

**Response:**

We are sorry for using a vague expression; we had copied the phraseology used by the participants. What we mean is that the participants felt that the government neglected healthcare in rural areas. We have updated the expression for clarity.

**Old text-**

They complained of stepmotherly treatment by the government.

**Modified text-**

They complained of being neglected by the government. (Page No: 17, Line No: 341–342)

d. Could you expand / reference examples of stigma regarding mental health issues in the healthcare community?

**Response:**

We have expanded the explanation of the stigma regarding mental health issues in the healthcare community, in the “**4.** **Discussion**” section

**Added text-**

Studies have shown that HCPs do not prefer availing help on mental health issues due to the stigma surrounding mental health in the healthcare community [4]. In a study of 11 doctors by Stanton & Randal [5], all participants, admitted to existence of a culture in which they are expected to be a super-person and that their reputation in the medical community could be harmed if they showed any vulnerability. This stigma possibly arises from the belief that ‘doctors are invincible’ and ‘illness is only for patients’ [4]. Galbraith et al. [6] studied the attitudes of student nurses toward stress and help-seeking, and found out that only 1.4% of the participants preferred revealing their mental health issues, and the others cited reasons like stigma (24.7%), fear of damage to professional integrity (14.6%), and fear of facing career implications (45.2%) for not revealing their mental health issues. Further, this study also expounded that the negative attitudes toward help seeking help on mental health issues may have been inculcated even before training [4]. (Page No: 31–32, Line No: 657–668)

6. Discussion

a. Consider providing more specific suggestions for future change based upon the detailed and novel findings of your study

**Response**:

Thank you for the suggestion. We have added various implications of the study under “**4. Discussion**” section. However, these are generic implications, as any specific future change can only be suggested through further studies employing quantitative methods to verify our findings. We have already initiated a study to empirically verify the efficacy of spiritual healing interventions on HCPs based on the results of this study.

**Added text-**

Subsequent studies, each founded on the detailed investigation of case studies, like this one, can add to these findings, in order to establish more general claims. The emphasis on the individual making sense of their mental health related issues lays foundation for a rich dialogue with quantitative models in mental health psychology. Sense-making is fundamental to various models used by health psychologists to examine individuals’ behaviours [7], and it is also central to how we have outlined the experience of the HCPs. Mixed method studies combining the findings of this experiential qualitative IPA study and that of the quantitative methods using standardized mental health screening tools such as the GAD-7, K-10, and DASS-21 can lead to a greater understanding of the HCPs’ mental health status, which in turn can be used to devise stress management policies that are tailored for the HCPs.

One of the key findings of this study is the use of spirituality as a coping mechanism by the HCPs; accordingly, we have devised a spiritual healing intervention to combat stress in HCPs and are running a clinical trial to assess the efficacy of this intervention.

This study demonstrates the shared themes of the participants while at the same time retaining the individuals’ experiences. By understanding a small number of individual cases in detail, we are in a better position to contemplate how, at a profound level, we share much in common with people whose circumstances may initially appear entirely different from our own. We hope that the readers can perceive the significance of individual accounts presented in this study in helping to comprehend what it is like to provide healthcare services during a pandemic in a resource-poor rural setting. Although the readers may not share the experience of these HCPs, we hope that they feel some sort of a resonance with the way it has impacted them existentially. (Page No: 33–34, Line No: 692–712)

**Comments from Reviewer#2:**

1. This is a very timely contribution to the body of literature related to healthcare workers’ perspectives during Covid-19. However below are some concerns which authors can address before this manuscript is considered for publication:

**Response**: Thank you for your kind evaluation of our work; we hope we have satisfactorily replied herein to all your questions and comments.

2. The authors used interpretative phenomenological analysis (IPA) but it lacks some link with the theoretical underpinnings of IPA. For instance, IPA is an idiographic approach, hence, the authors need to present the results by illuminating an individual healthcare worker’s perspective in addition to the group themes currently presented, in order to alien the result with IPA’s individualistic/idiographic approach. It is highly recommended to consult the latest/revised terminologies of results/themes in an IPA study i.e., Personal experiential themes and group experiential themes. Full details of the new terminology and worked examples of their use in practice are provided in the new books:

   Smith JA , & Nizza I (2021) Essentials of Interpretative Phenomenological Analysis. Washington DC: APA.

   Smith JA, Flowers P, Larkin M (2022) Interpretative Phenomenological Analysis: Theory, Method, Research. London: Sage by Smith

   However, if the authors do not want to use the new terminologies, still it is recommended to highlight at least the idiographic focus in the result section.

**Response**:

We agree with the reviewer’s suggestions. We have attached an appendix of the unedited excerpts (along with annotations of non-verbal cues) that underpin this analysis. These excerpts throw light on the individual perspectives of the participants.

The second part of this comment recommends the usage of latest terminologies. We tried to find the online copies of the 2 books recommended, but with no luck. We found older editions of 2009. The ones published in 2021 & 2022 were not available. Paucity of time made it impossible to order physical copies, which would have taken 3–4 weeks to reach. Since it was an optional requirement to add the latest terminologies, we decided to carry on with the present terminologies.

3. Furthermore, the authors need to also inculcate the hermeneutic and double hermeneutic stance of IPA in this study by not just descriptively presenting their own opinion after each theme but go deeper in the analysis and present descriptive, linguistic and conceptual analysis of each theme.

**Response**:

We agree with the reviewer. Accordingly, we have added the subsection **“3.2 Investigation of individual themes”** in the “**3. Results**” section, wherein we present a deeper analysis of the participants’ experiences during the pandemic using a hermeneutic and double hermeneutic approach. However, considering the length of the overall manuscript, we decided to keep this brief for each participant.

**Added text-**

***3.2 Investigation of individual themes***

All significant utterances of each participant that underpinned the analysis are presented in the S1 Appendix. The subsequent subsections briefly present the individual themes of each participant.

**3.2.1 Varun**

Varun provides the perspective of a senior critical care specialist. He has made sense of the situation as being in a war and he sees himself as a soldier in this war. When talking about the pandemic, he repeatedly mentioned the risk to his husband, while alluding to his own risk of contracting COVID-19 only once, that too when talking about the risk to HCPs in general. He is not satisfied with the standard of care he is providing because the hospital he works for has restricted cardiopulmonary resuscitation in COVID-positive patients given the virus-aerosolization risk, and because he is unable to explain the deaths of his patients. He is afraid of missing some potential treatment, so he reads recent research and treatment guidelines and follows them diligently. Unless he has tried every measure to save his patients, he feels morally conflicted. He feels that his exposure to COVID-19 is a given and is willing to go to great lengths to save others, even at a great personal cost. A sense of frustration at the uncertainty of the pandemic, high cost of treatment, and the careless attitude of the public is palpable from his account of the pandemic and his non-verbal cues. His attitude towards his work and general outlook during the pandemic appears to be consistent with that of a soldier at war, willing to make the supreme sacrifice.

**3.2.2 Vayu**

Vayu provides the perspective of a first responder. He repeatedly mentioned engaging in frontline COVID-19 work due to financial constraints. If he did not have a family to provide for, he would not work as a frontline HCP as he is afraid of contracting COVID-19 given his history of diabetes; thus, he has placed the needs of his family above his own needs. At one instance, he narrated an incident where he did not remove an abusive individual from his ambulance because this individual’s mother—the patient—required oxygen support. He felt that the patient would not be comfortable without her son; he displayed empathy towards his patient and put her needs above his own need to remove himself from an abusive situation. Although he has made sense of his engaging in work during the pandemic as a compulsion due to financial needs, he also seems to derive a sense of self from his work as a paramedic; he lost his sense of self during the pandemic due to witnessing the deaths of patients owing to a shortage of beds and oxygen and his helplessness in the face of COVID-19.

**3.2.3 Sani**

Sani provides the perspective of a doctor who is not trained in managing acute respiratory diseases but had to treat severe cases of COVID-19 during the second wave of the pandemic in India. She repeatedly mentioned her lack of training throughout her account. She feels she is not the right person to be treating severe cases of COVID-19 and is questioning her decision to volunteer for frontline COVID-19 work. Deaths of patients due to drug shortages adversely affected her mental health; in a way she feels responsible for those deaths. She repeatedly mentioned God during the interview and is drawing strength from her spiritual practices to deal with the pandemic and with her doubts about her ability to treat severe cases of COVID-19. She has made sense of her experience of the pandemic as someone who made an ill-informed wrong choice, albeit an altruistic one, and is doing her best to avoid its negative consequences on the wellbeing of others.

**3.2.4 Bhumi**

Bhumi provides the perspective of a senior nurse. She repeatedly mentioned a lack of financial reward for her work during the pandemic. She is dissatisfied with the compensation she is receiving for her work, but she feels a strong sense of belonging with the community of HCPs, which motivates her to continue working. Lack of acknowledgement from patients for her contribution to their recovery has adversely affected her self-worth. She has not yet made sense of her experience of the pandemic, and is mechanically going about her life and work. She feels that if she reflects on her experiences and tries to make sense of them, she will not be able to cope; she has decided to put off this exercise until the end of the pandemic and concentrate on completing the work that is assigned to her. Interestingly, although she feels close to her family, she was the only participant who did not mention family at all during the interview, other than a passing reference to her husband. This may be because she has not yet reflected on how the pandemic is affecting her, or because she has compartmentalised her life and sees COVID-19 as only a work-related problem.

**3.2.5 Agni**

Agni provides the perspective of an allied health worker who is not trained to manage lung diseases but had to manage severe cases of COVID-19 during the second wave of the pandemic in India. Unlike Sani, she did not question her decision to volunteer. In fact, she had a positive attitude towards her decision to volunteer and regardless of training, she did not feel someone else could have done a better job than her. She felt a strong sense of responsibility towards her patients, from which she drew motivation to continue working during the pandemic. The uncertainty surrounding COVID-19 was a recurrent motif in her narrative; it threatened her sense of safety. A threatened sense of safety severely impacted her quality of life. The meaning she attached to the pandemic was that of a poorly-understood phenomenon that is to be feared. She felt disconnected from her family as she felt they do not share her experience of the pandemic. She felt only her peers could understand her perspective, but due to stigma and fear of appearing weak in front of her peers, she did not discuss her fears with them. (Page No: 9–12, Line No: 179–248)

4. The following statement in the result section of the abstract needs to be rephrased to bring more clarity “Our findings demonstrate that the participants were mental duress due to heavy workloads”.

**Response**:

Thank you for pointing this out. We have rephrased the aforementioned statement to bring more clarity.

**Old text-**

“Our findings demonstrate that the participants were mental duress due to heavy workloads”

**Modified text-**

“Our findings demonstrate that the participants were under mental duress due to heavy workloads” (Page No: 2, Line No: 32–33)

5. Also, start the result section in the abstract by presenting the total number of super-ordinate and subordinate themes and their labels.

**Response**:

Thank you for the suggestion. We added the total number of master themes and subthemes to the “**Results**” sub-section of the “**Abstract**” section. However, due to the word count restrictions in the abstract, we were unable to add the labels of the master themes and subthemes.

**Added text-**

Six master themes and 23 subthemes were identified. (Page No: 2, Line No: 32)

5. In the method section, need to elaborate more how IPA is the best suited method in this study with more citations.

**Response**

Thank you for the suggestion. We have added the justification for selecting IPA as a method for the study under section **“1. Introduction”**

**Added text-**

Since the start of the pandemic, we have collaborated with the public health authorities of Dhanbad, India to investigate whether the frontline workers in different settings of the district could be supported in any way in their fight against COVID-19; as part of this endeavour, this study aimed to investigate the lived experiences of the participants and at the same time capture any generalisation, if discernible. Due to the enormous stress experienced by the HCPs during the COVID-19 pandemic [8, 9], we aimed to use the results of the current study and other completed and ongoing studies resulting from our collaboration with the public health authorities of Dhanbad, India, to subsequently design an intervention to mitigate stress and tailor it in such a way as to maximise its use by the frontline workers of the COVID-19 pandemic in Dhanbad, India. We believe that it is vital to take notice of individual accounts in order to determine effective interventions tailored to the population of interest; therefore, a qualitative method was considered to be best suited to achieve this goal. Among the main methods of qualitative research (phenomenology, grounded theory, discourse analysis and narrative analysis), the phenomenological approach was preferred as the study is concerned with understanding the experience of a particular phenomenon (delivering healthcare during the COVID-19 outbreak). As the study aims to keep one eye on the individual accounts, while keeping another eye on any possible generalisation—which can be achieved by an analysis of the convergence and divergence of individual accounts—within the phenomenological approaches, IPA was chosen as IPA shows both convergence and divergence between participants, while retaining their individuality [7]. Nomothetic methods were rejected as they are probabilistic, providing group averages wherein individual experience is lost [10]. (Page No: 4–5, Line No: 74–94)

6. Furthermore, authors need to explain within their data collection section (with citations) why unstructured interviews were used whereas in IPA the semi-structured interviews are highly recommended.

**Response**:

Thank you for the suggestion. We have added the justification for the usage of unstructured interviews, under subsection “***2.3 Setting and data acquisition”.***

**Added Text-**

Unstructured interviews were preferred over semi-structured ones as they offer an opportunity to capture the experience of the interviewee in the most concrete form possible [11, 12], which is central to the phenomenological underpinnings of IPA. In addition, the unstructured nature of the interviews allows exploration of unanticipated paths which reflects the inductive epistemological position of IPA [13]. Moreover, the unstructured method also helps reduce the status difference between the interviewer and the interviewee [12]. Additionally, in unstructured interviews, the interviewee is central to the process and commands most of the control of the process and content [14; 15]. Therefore, we opined that the HCPs, who were under enormous stress during the pandemic [8; 9], would feel much more at ease during the interviews, and the interview process might even provide a therapeutic benefit by giving them an opportunity for catharsis [11]; thereby aligning with the goals of our collaboration with the public health authorities of Dhanbad, India. (Page No: 6–7, Line No: 134–145)

7. Add some research, policy, and clinical implications in the discussion section

**Response**:

Thank you for the suggestion. We have added various implications of the study under “**4. Discussion**” section. However, these are generic implications, as any specific future change can only be suggested through further studies employing quantitative methods to verify our findings. We have already initiated a study to empirically verify the efficacy of spiritual healing interventions on HCPs based on the results of this study.

**Added text-**

Subsequent studies, each founded on the detailed investigation of case studies, like this one, can add to these findings, in order to establish more general claims. The emphasis on the individual making sense of their mental health related issues lays foundation for a rich dialogue with quantitative models in mental health psychology. Sense-making is fundamental to various models used by health psychologists to examine individuals’ behaviours [7], and it is also central to how we have outlined the experience of the HCPs. Mixed method studies combining the findings of this experiential qualitative IPA study and that of the quantitative methods using standardized mental health screening tools such as the GAD-7, K-10, and DASS-21 can lead to a greater understanding of the HCPs’ mental health status, which in turn can be used to devise stress management policies that are tailored for the HCPs.

One of the key findings of this study is the use of spirituality as a coping mechanism by the HCPs; accordingly, we have devised a spiritual healing intervention to combat stress in HCPs and are running a clinical trial to assess the efficacy of this intervention.

This study demonstrates the shared themes of the participants while at the same time retains the individuals’ experiences. By understanding a small number of individual cases in detail, we are in a better position to contemplate how, at a profound level, we share much in common with people whose circumstances may initially appear entirely different from our own. We hope that the readers can perceive the significance of individual accounts presented in this study in helping to comprehend what it is like to provide healthcare services during a pandemic in a resource-poor rural setting. Although the readers may not share the experience of these HCPs, we hope that they feel some sort of a resonance with the way it has impacted them existentially. (Page No: 33–34, Line No: 692–712)

**References:**

1. Census2011.co.in. 2022. Indian Districts by Population, Sex Ratio, Literacy 2011 Census. [online] Available at: <https://www.census2011.co.in/district.php> [Accessed 17 April 2022].
2. DailyRounds. 2022. *This state last got a 'new' medical college 50 years back! - DailyRounds*. [online] Available at: <https://www.dailyrounds.org/blog/this-state-last-got-a-new-medical-college-50-years-back/> [Accessed 17 April 2022].
3. Legard R, Keegan J, Ward K. In-depth interviews. In: Ritchie J, Lewis J, editors. *Qualitative Research Practice: A Guide for Social Science Students and Researchers.* London: Sage; 2003. pp. 139–169
4. Henderson M, Brooks SK, del Busso L, Chalder T, Harvey SB, Hotopf M, et al. SHAME! self-stigmatisation as an obstacle to sick doctors returning to work: A qualitative study: Table 1. BMJ Open. 2012;2(5).
5. Stanton J, Randal P. Doctors accessing mental-health services: an exploratory study. 2011.
6. Galbraith N, Brown K, Clifton E. A Survey of Student Nurses' Attitudes Toward Help Seeking for Stress. Nursing Forum. 2014;49(3):171-181.
7. Smith J, Flowers P, Larkin M. Interpretative phenomenological analysis. London: SAGE; 2009.
8. Chen Q, Liang M, Li Y, Guo J, Fei D, Wang L et al. Mental health care for medical staff in China during the COVID-19 outbreak. The Lancet Psychiatry. 2020;7(4):e15-e16.
9. Duan L, Zhu G. Psychological interventions for people affected by the COVID-19 epidemic. The Lancet Psychiatry. 2020;7(4):300-302Brocki JM, Wearden AJ. A critical evaluation of the use of interpretative phenomenological analysis (IPA) in health psychology. Psychology and Health. 2006;21(1):87-108.
10. Datan N, Rodeheaver D, Hughes F. Adult Development and Aging. Annual Review of Psychology. 1987;38(1):153-180.
11. Amos IA. Attempting to capture the ineffable quality: An interpretative phenomenological analysis and embodied interpretation of the experience of sudden personal transformation. The University of Manchester. 2026 [cited 17 April 2022]. Available from: https://www.research.manchester.ac.uk/portal/files/57430681/FULL_TEXT.PDF
12. Finlay L. The body's disclosure in phenomenological research. Qualitative Research in Psychology. 2006;3(1):19-30.
13. Brocki J, Wearden A. A critical evaluation of the use of interpretative phenomenological analysis (IPA) in health psychology. Psychology &amp; Health. 2006;21(1):87-108.
14. Morse JM. Interviewing the ill. In Gubrium J, Holstein J editors. Handbook of Interview Research: Context and Method. Thousand, CA: Sage; 2002. pp. 317–328.
15. Fontana A, Frey JH. The interview: From structured questions to negotiated text. In: Denzin NK, Lincoln YS editors. Handbook of qualitative research. 2nd ed. Thousand Oaks, CA: Sage; 2000; pp. 645-672.
