# Supplementary material for: Experiencing the COVID-19 pandemic as a healthcare provider in rural Dhanbad, India: An interpretative phenomenological analysis
Source: PLoS One. 2022 Aug 25;17(8):e0273573. doi: 10.1371/journal.pone.0273573 (PMC9409569; doi:10.1371/journal.pone.0273573)
Supplement: Attachment — Submitted filename: Response to Reviewers.docx [file pone.0273573.s003.docx]

We would like to thank the Reviewer for judging our work positively and the Academic Editor for the insightful comments that have helped us further polish our manuscript. Below are our point-by-point responses to the questions and comments raised during his review. We sincerely hope that we have satisfactorily responded to all the queries and look forward to hearing back soon.

Please note: The page and line numbers mentioned in this document pertain to the manuscript with changes tracked, in the all-markup view.

| **S. No.** | **Academic Editor’s Comments** | **Authors' Responses** |
| --- | --- | --- |
| 1 | Line 46. Update COVID stats to be more recent | Thank you for your comment. We have updated line 46 with latest COVID stats. |
| 2 | Line 66 To date not Till date | Thank you for your suggestion. We have modified "Till date, very few studies" to "To date, very few studies" in line 66. |
| 3 | Line 87. Ensure the meaning of the IPA abbreviation is spelt out on first mention, not subsequent mentions (currently line 96). | Thank you for pointing this out. We have now mentioned the full form of the abbreviation IPA at its 1st appearance (line 87), and have removed its expanded notation from line 97. |
| 4 | Line 90 Aims to, not attempts to | We have modified "this study attempts to" to "this study aims to" in the manuscript. Now it is at line 91. |
| 5 | Line 178. Varun is described as male and having a husband. As no other mention of him being in a same-sex relationship was evident in my reading, I wonder if “marital partner” might be a better term to use here, to save the reader from wondering whether a same-sex relationship was relevant to the experience of the pandemic. | Although being in a same-sex relationship in rural India (where such relationships are taboo and only recently decriminalised) places additional burdens on mental resilience and may thereby be of relevance to the experience of the pandemic, we have not explored this aspect in our study. Therefore, we agree with your suggestion and have changed “husband” to “marital partner”. This can be found at line 179. For the sake of consistency, we have also made this change at line 227. |
| 6 | Line 194 Is history of diabetes appropriate? In my lay understanding is a chronic condition that is managed, so wouldn’t this participant still have diabetes? I would have thought “diabetic status” is appropriate, but please use whatever description you think is most accurate. | Although doctors routinely use “history of diabetes” when referring to a background of having diabetes while talking about a patient, especially during handover of care, we agree that it may cause confusion to non-medical professionals. Therefore, we have changed “history of diabetes” to “diabetic status” in line 196. |
| 7 | Line 226-228 Sentence beginning “This may be because…” seems to be very speculative to me, as it is based on what the participant does not say rather than what they said. Please consider deleting it. | Thank you for your pointing this out. It is indeed an interpretation of the researcher of the things not said by the participant. We agree that it is speculative and have deleted the sentence (Line 227–229). |
| 8 | Table 2, theme 1. Please provide an alternative term to “loot” as I am not sure what that would mean in this context. Having read further, I think “financial exploitation” might be clearer. Or perhaps profiteering. | Thank you for your suggestion. We have modified "loot" to "profiteering" in the table 2, theme 1 of the manuscript |
| 9 | Table 2, theme 3. The justification on the theme name seems odd, please re-space. | Thank you for pointing this out. We have now changed the justification of theme 3 |
| 10 | Line 384 Define aerosolization for a non-specialist audience | We have added an explanation in line 183 where the term is first mentioned. |
| 11 | Line 402 Some reason or other (delete “the”) | We have modified "some reason or the other" to "some reason or other". Now it is in line 403. We have also made the same change at line 294. |
| 12 | Line 528 Off from not Off off | We have modified "minds off of" to "minds off from". Now it is in line 529. |
| 13 | Line 586 Highlighted the (delete “of”) | Thank you for pointing this out. We have modified "participants highlighted of the need" to "participants highlighted the need". Now it is in line 587. |
| 14 | Line 594-664 This paragraph is too long and needs to be broken up. | We have introduced paragraph breaks at lines 605, 616, 626, 643, and 650. |
| 15 | Line 659 help is repeated. | Thank you for pointing this out. We have modified "attitudes toward help seeking help" to "attitudes toward seeking help". Now it is in line 664. |
| 16 | Line 693-695 This paragraph is not sufficiently developed as indicated by it forming only a single sentence. I recommend developing it or removing it. | We agree with your observation and have accordingly merged the information in this paragraph with a previous paragraph discussing our findings on spirituality. The paragraph has been deleted from lines 699–701 and the sentence has been added to lines 669–670. |
| 17 | Line 705 has caused A significant amount… (insert A) | We have modified "pandemic has caused significant amount" to "pandemic has caused a significant amount". Now it is in line 711. |
